# Intestinal Disaccharidase Deficiency in Adults: Evaluation and Treatment

**DOI:** 10.1007/s11894-023-00870-z

**Published:** 2023-05-18

**Authors:** Lavanya Viswanathan, Satish SC Rao

**Affiliations:** 1 Department of Gastroenterology, Hepatology and Motility, David Grant Medical Center, Travis Air Force Base, 101 Bodin Cir, Travis AFB, CA 94535 USA; 2 Uniformed Services University of the Health Sciences, University of California Davis, Davis, USA; 3 Medical College of Georgia, Augusta University, Augusta, USA

**Keywords:** Disaccharidase, Sucrase Isomaltase Deficiency, Carbohydrate malabsorption

## Abstract

**Purpose of Review:**

Disaccharidase deficiency in adults causes carbohydrate malabsorption, resulting in symptoms which significantly overlap with irritable bowel syndrome (IBS). This article discusses the diagnosis and treatment of disaccharidase deficiency within the context of recent literature.

**Recent Findings:**

Disaccharidase deficiency in adults is more common than previously thought, which includes lactase, sucrase, maltase and isomaltase enzymes. Deficiency in disaccharidases, which are produced by the intestinal brush border, will interfere with the breakdown and absorption of carbohydrates and may result in abdominal pain, gas, bloating and diarrhea. Patients deficient in all 4 disaccharidases are known as having “pan-disaccharidase” deficiency, which has a distinct phenotype with more reported weight loss than patients deficient in one enzyme. IBS patients who do not respond to low FODMAP dietary restriction may have undiagnosed disaccharidase deficiency and may benefit from testing. Diagnostic testing methods are limited to duodenal biopsies, which is the gold standard, and breath testing. Dietary restriction and enzyme replacement therapy have been shown to be effective treatments in these patients.

**Summary:**

Disaccharidase deficiency is an underdiagnosed condition in adults with chronic GI symptoms. Patients who do not respond to traditional treatment strategies for DBGI may benefit from testing for disaccharidase deficiency. Further studies delineating the distinctions between disaccharidase deficient patients and those with other motility disorders are needed.

## Introduction

The implications of disaccharidase deficiency as it pertains to neurogastroenterology and motility disorders has been a mystery for the past several decades. While congenital disaccharidase deficiency has been established as a cause of chronic abdominal pain in young children, it is more common in adults than previously thought [1, 2]. This review serves to summarize what is known thus far about disaccharidase deficiency in adults, how to evaluate adult patients for such deficiencies and treatment strategies.

## Carbohydrate Digestion and DGBI

As carbohydrates comprise a large proportion of daily caloric consumption, the problem of carbohydrate intolerance is all the more significant due to the rising consumption of sugars by Americans [1, 3−5]. The average American has a daily carbohydrate intake of 300 g and sugar intake of 140 g [6]. Lactose and sucrose are the most common disaccharides found in the diet [7]. Lactose is the main sugar in milk, and lactase non-persistence is the common phenotype in healthy humans, which accounts for the downregulation of lactose after infancy [7]. Diarrhea usually results if the load of lactose exceeds the colonic capacity for resorption but this is also dependent upon the intestinal microbiome, microbial function and small bowel bacterial overgrowth [8]. Lactase deficient patients with IBS also appear to express more severe symptoms associated with lactose intake, so visceral hypersensitivity also plays a role [8].

Sucrase is critical in metabolizing table sugar to glucose. The Western diet is high in sucrose, which is present in fruits such as peaches and cherries, desserts and sugary drinks [9]. Sucrase-Isomaltase (SI) gene product is located along the mucosal brush border which cleaves isomaltase and sucrose into sugar monomers. These cleaved monosaccharides are then transported across the epithelial brush border for absorption and metabolism [10]. Certain variants of congenital SI deficiency (CSID) result in sucrose and starch intolerance. CSID is an autosomal recessive disorder of carbohydrate metabolism involving the Sucrose-Isomaltase (SI) gene. CSID mutations are more commonly found in IBS patients vs. healthy controls, and symptom expression is heterogenous depending on the biochemical phenotype of the mutation [11, 12].

**Fig. 1:**
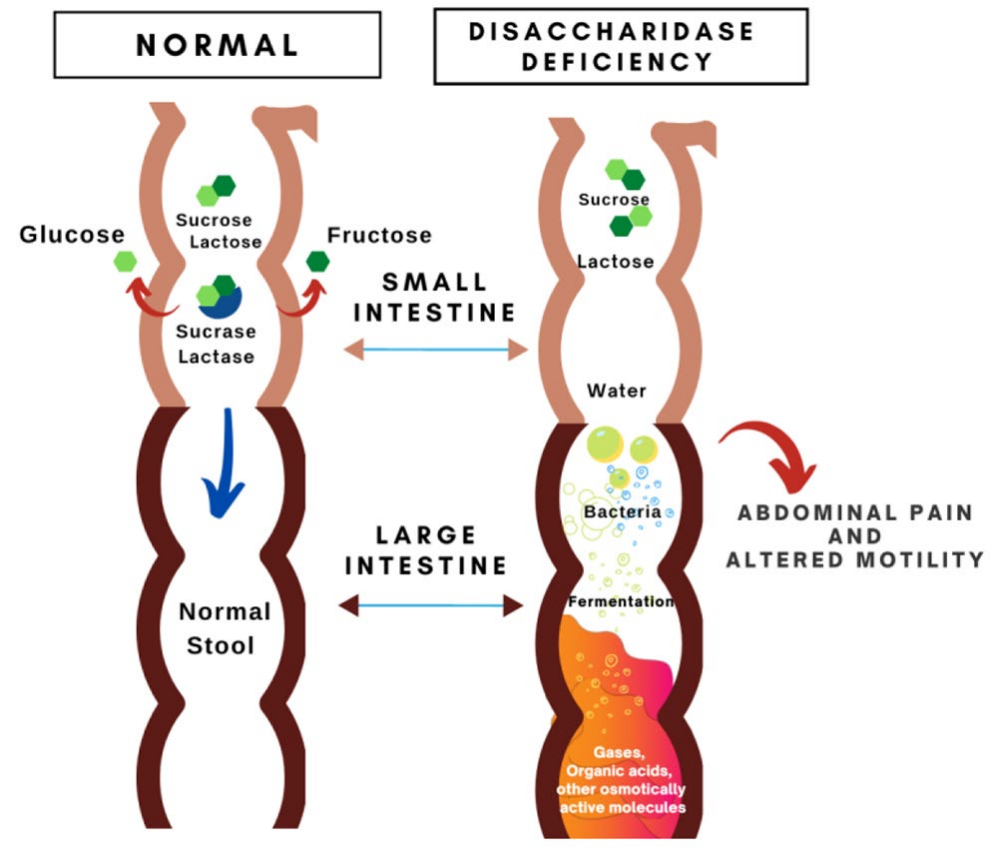
Effect of fermented starches in disaccharidase deficient patients compared to normal, healthy patients

Carbohydrates are broken down in large part by disaccharidases found along the brush border of the small intestine into monosaccharides such as glucose, galactose and fructose which are transported across the epithelial brush border where they are absorbed and metabolized. The disaccharidases lactase, sucrase, maltase and isomaltase (also referred to as palatinase or trehalase) serve to break down sugars into monosaccharides, allowing for quick absorption [13]. Disaccharidase activity varies depending on location and is highest in the mid jejunum and lower in the proximal duodenum and terminal ileum [14]. A deficiency in any one disaccharidase can result in increased osmotic load from malabsorbed sugars in the small intestine (Fig. 1). This can result in abdominal pain, gas, bloating and diarrhea which are symptoms commonly reported by patients with Disorders of Gut-Brain Interaction (DGBI) [15, 16]. Diets low in fermentable substrates are associated with improved symptoms in Irritable Bowel Syndrome (IBS) patients [17]. A diagnosis of IBS may miss potential disaccharidase deficiencies, so earlier diagnosis of disaccharidase deficiencies may lead to more successful treatment of DGBI.

## Disaccharidase Deficiency in Adults

**Fig. 2:**
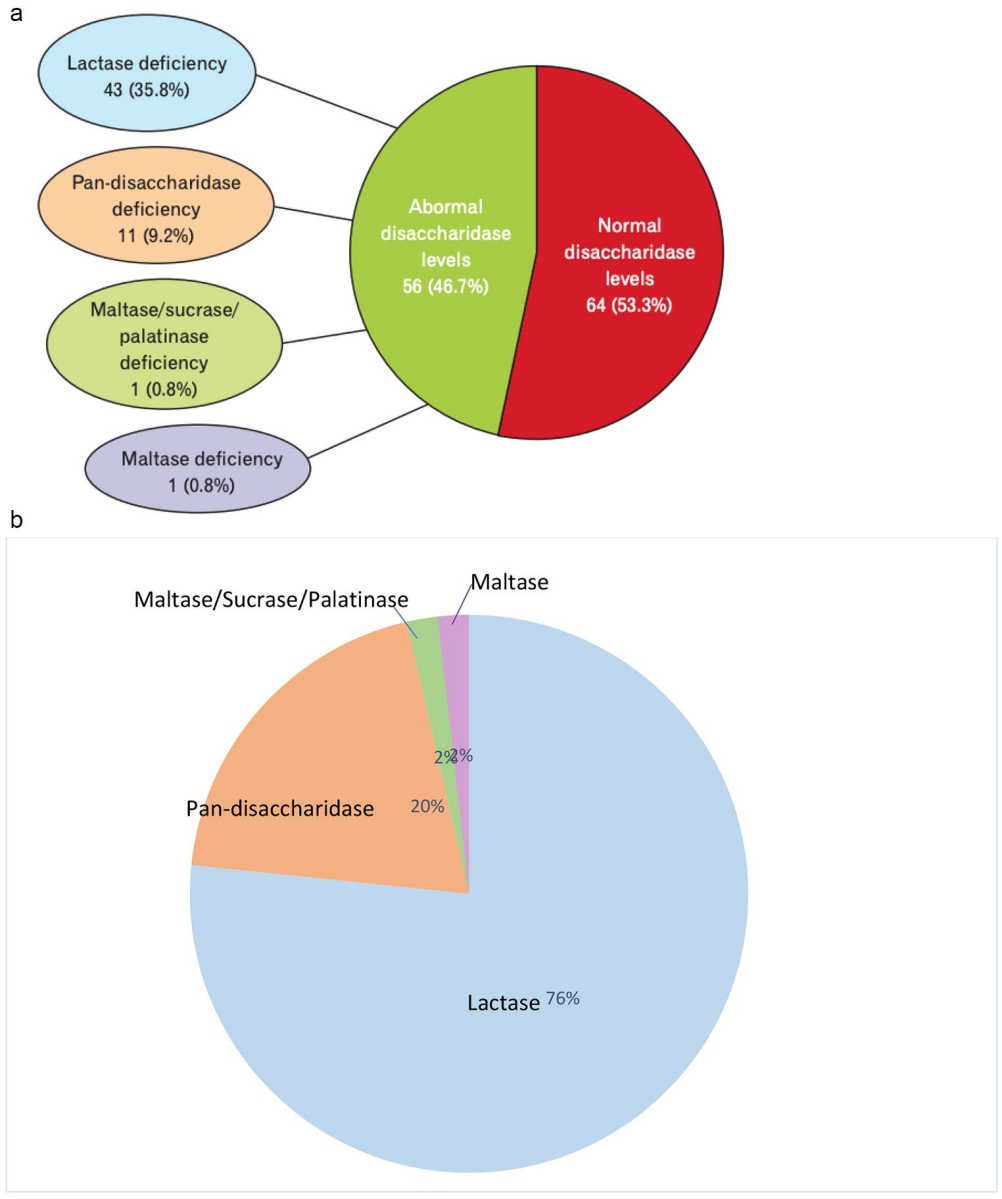
(**a**) Prevalence of normal and abnormal disaccharidase levels in adult cohort [2]. *Reprinted from J Neurogastroenterol Motil 2020; Vol. 26, No. 3, Page 387 with permission*. (**b**) Proportion of abnormal disaccharidase levels in adults [2]

The incidence of sucrase-isomaltase deficiency has been estimated to be between 0.2% in North America and 10.0% in Greenland Eskimos [18]. Disaccharidase deficiencies have been well documented in children to explain abdominal pain. Prevalence of disaccharidase deficiencies within the pediatric population has been reported to be 37% lactase, 25% maltase, 21% sucrase and 8% palatinase [1]. Cases of children with all 4 disaccharidase deficiencies have not been reported in the literature. *Viswanathan et al.*. reported the first published prevalence data of disaccharidase deficiency in 120 adult patients with unexplained gastrointestinal symptoms. They found that 9.2% of patients had all 4 disaccharidase enzyme (pan-disaccharidase) deficiencies, 35.8% were lactase deficient, 0.8% were maltase deficient and 0.8% had combined sucrase, maltase and isomaltase deficiency [2] (Fig. 2). Figure 2b shows the proportional distribution of disaccharidase deficiencies in adults. Patients with single disaccharidase deficiency reported abdominal pain, bloating, fullness, gas, indigestion, cramping and nausea and reported less vomiting and weight loss [2]. The pan-disaccharidase cohort reported bloating and constipation, but reported relatively less cramping, pain, fullness, nausea, belching, diarrhea and gas, though these data were not significant [2]. Interestingly, they also reported more weight loss as compared to the single disaccharidase deficiency group [2]. This suggests that disaccharidase deficiencies occur often in combination, pan-disaccharidase deficiency is more common in adults than previously thought and this cohort of patients has a distinct clinical phenotype. Further characterization of the pan-disaccharidase group is needed, including possible etiology and its relationship with dysbiosis.

IBS affects 10% of the Western population, and its associated symptoms can mimic the post-prandial symptoms related to carbohydrate intake reported in disaccharidase deficient patients [18]. Thirty-one patients with presumed IBS-D/M based on symptoms of pain, bloating and diarrhea underwent disaccharidase assays [15]. Sucrase-isomaltase deficiency (SID) was present in 35% of patients, and these patients were less likely to report abdominal pain [15]. No difference in bloating or diarrhea was found between SID and IBS-D/M patients [15]. SIBO was not excluded in these 31 patients, which may have overestimated the prevalence of IBS. In another study of 82 patients with a mix of functional diarrhea and constipation, a majority of patients expressed disaccharidase deficiency: 86.5% were lactase deficient, 48.7% were maltase deficient, 50% were sucrase deficient and 84.1% were gluco-amylase deficient [19]. Interestingly, 31.7% of these patients were deficient in all enzymes [20].

## Testing

**Fig. 3:**
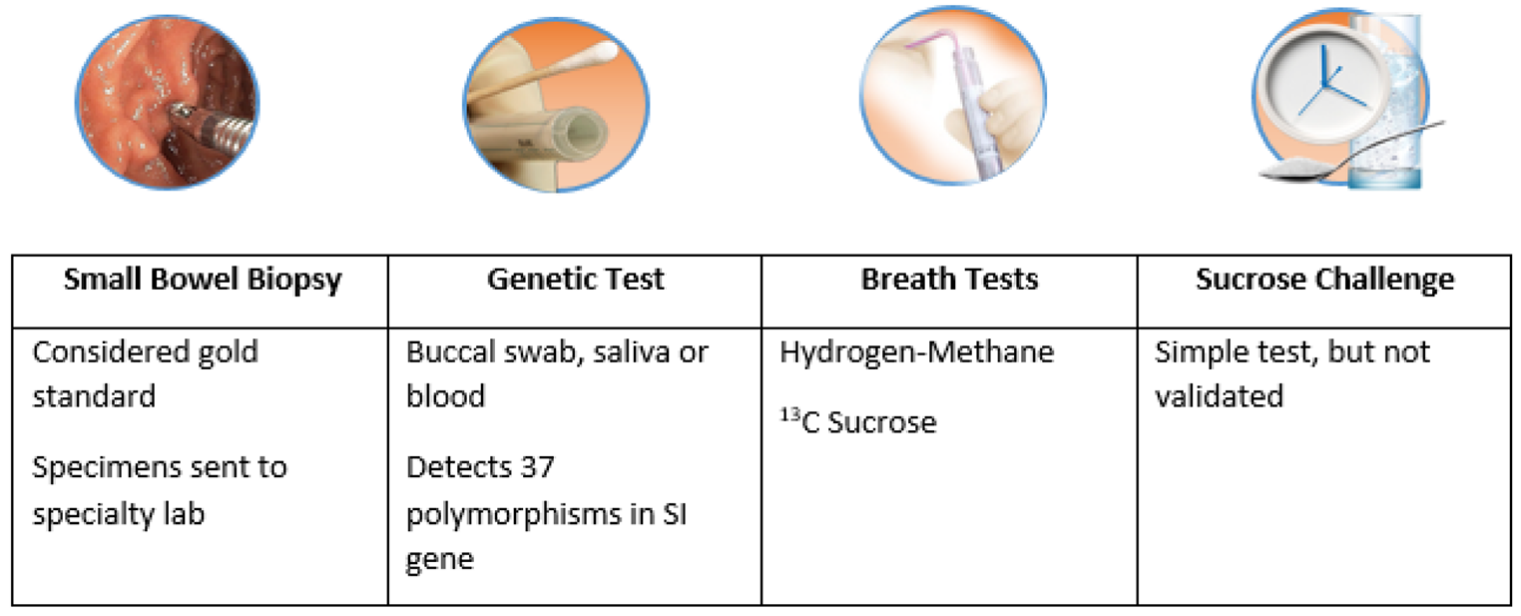
Tests which aid in diagnosing CSID [10, 23, 28]

Testing options for the diagnosis of disaccharidase deficiency include disaccharidase assay, genetic testing, breath testing and disaccharide challenge (Fig. 3). The diagnosis should be considered in patients complaining of lifelong postprandial abdominal pain, gas, bloating and/or diarrhea. Secondary causes of disaccharidase deficiency, such as Celiac or Crohn’s disease, chemoradiation therapy, small intestinal bacterial overgrowth (SIBO), acute gastroenteritis or any other condition which may damage the brush border, should be excluded.

**Fig. 4:**
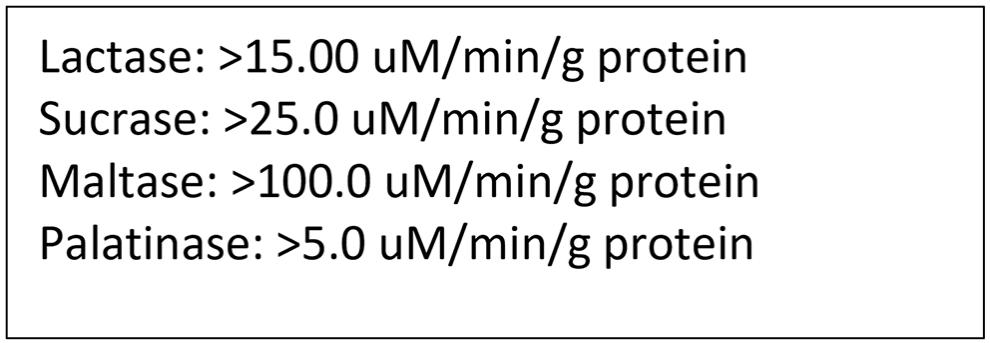
Reference levels of normal disaccharidase levels from duodenal biopsies in adults

The gold standard for testing disaccharidase deficiency is endoscopically obtaining two biopsies from the third portion of the duodenum [2, 15]. One biopsy is used to study the architecture of the mucosa and the second is used for analysis of sucrase, maltase, lactase and isomaltase levels per the Dahlqvist method. This method gives precise levels of disaccharidase levels; however, it can be limited by sample error, as two biopsies may not give a complete picture of brush border enzyme activity as enzyme distribution can be patchy [12]. Biopsies from distal duodenal provide higher yield than those from the proximal duodenum [14]. Figure 4 shows normative data of disaccharidase levels from duodenal biopsies in adults per the Dalqvist method. Current reports of disaccharidase assays from commercial labs also include alpha-glucosidase and alpha-amylase levels.

Sucrose hydrogen breath testing is readily available both as in clinic or at home options and is a non-invasive surrogate method for testing for disaccharidase deficiency. This test can be performed either by ingesting a standard amount of ^13^ C-sucrose or sucrose (known as a direct sucrose challenge) and measuring the amount of expired ^13^ C-methane or hydrogen, respectively [21]. ^13^ C-methane breath testing measures sucrase activity without administering a sucrose load to the patient while the direct challenge sucrose test is cheaper but will provoke symptoms. Breath test results correlate well with intestinal sucrase activity [12]. However, it is important to note that in practice, SIBO should be ruled out as the presence of bacterial overgrowth can confound sucrose breath test results [22]. False positive results can also be a result of villous injury, such as Celiac disease and dumping syndrome [23]. A study of 258 adults with chronic GI symptoms who underwent both hydrogen-methane and ^13^ C sucrase breath testing reported positive yield for ^13^ C sucrase breath test of 26% [24]. The hydrogen-methane group had false positives due to underlying SIBO [24]. Patients with sucrose deficiency identified on ^13^ C sucrose breath testing reports functional diarrhea, flatulence, bloating and IBS-M and 60% of those patients reports improvement with dietary adjustment and/or enzyme replacement therapy [24]. Symptom severity is dependent upon several factors, to include sucrase-isomaltase activity, amount of sugar and starch intake and gastric and small bowel transit [23]. While all CSID patients have sucrase deficiency, they vary in degree of isomaltase activity [25]. Therefore, patients with disaccharidase deficiency can present with a varied constellation of symptoms so it is important to keep this diagnosis on your differential in the face of chronic, nonspecific GI symptoms.

## Treatment

Patients with IBS-D with underlying SI deficiency did not experience any benefit from a low FODMAP (fermentable oligosaccharide, disaccharide, monosaccharide and polyols) diet when compared with non-SI carriers [26]. This may be because, in patients with SI, sucrose is metabolized as a FODMAP and fermented in the colon. Dietary exclusion of sucrose and/or starches can be helpful, though difficult to continue longterm. Given the difficulty of adherence to this diet, patients may benefit from formal consultation with a GI dietician, if available.

Enzyme supplementation, either with lactase or sacrosidase is another treatment option. It is unknown whether treatment of lactase deficiency will improve symptoms of other disaccharidase deficiencies. Sacrosidase, sucrose enzyme derived from *Saccharomyces cervisiae*, is the only FDA approved treatment for CSID/SI patients though there are other over the counter options as well (Table 1). Sacrosidase treatment in pediatric patients improved symptoms of diarrhea, gas, cramping and bloating at a diluted dose and completely resolved symptoms at the full dose [23]. Additionally, sacrosidase oral replacement normalized ^13^C-sucrose breath test results in deficient patients [10, 12]. Similar results of improved symptoms of abdominal pain, bloating, flatulence, borborygmi and diarrhea and lower hydrogen breath test scores were reported by lactase deficient patients receiving lactase supplementation [27].

**Table 1:** Available treatment options for disaccharidase deficient patients

Name	Components	Cost	FDA Approved
Similase	Sucrase 300 IU Lactase 4,670 IU Amylase 32,000 USP Maltase 32,100 DP Protease 30,000 USP	$0.29 per pill	N
Starchway (Intoleran)	Sucrase 7,500 IU Glucoamylase 2,500 IU	$1.30 per pill	N
Baker’s Yeast	Sucrase 7,500 IU	$0.60 per ounce	N
Sacrosidase (Sucraid)	Sacrosidase 8,500 IU	$37.01 per mL	Y

Several other enzyme supplements are available over the counter, including vegan enzyme options, but these have not been evaluated in clinical trials. A recent product, FODZYME (inulinase enzyme powder), is now available over the counter, and is believed to break down fructans and other disaccharides. But its usefulness merits a randomized controlled trial.

## Conclusions

Disaccharidase deficiency is an important and underappreciated clinical problem in the adult population although more readily diagnosed in pediatric patients. This is a disease entity which hides in plain sight but may often be overlooked in patients labeled with IBS. Awareness and education about the importance of carbohydrate malabsorption will benefit both the clinician and the patient, especially in the context of unexplained, chronic, post-prandial symptoms of abdominal pain, gas, bloating and diarrhea. It should be considered in patients who have longstanding symptoms which are unresponsive to a low FODMAP diet or to other common treatments for IBS. The gold standard assay for testing disaccharidase function involves an esophagogastroduodenoscopy with 2 biopsies from the third portion of the duodenum but can be limited by sample error. Less invasive surrogate tests include the direct sucrose challenge and the ^13^C-sucrose breath test. SIBO should be exclude first, when employing breath testing to avoid a false positive result. Once the diagnosis is confirmed, treatment options include dietary restrictions vs. enzyme replacement therapy.

While we now know more about this disease entity, there is much more to be discovered. Larger population studies are needed, as this epigenetic phenomenon is expressed differently, depending on the patient. Clinical stratification to predict who may develop disaccharidase deficiency and who may benefit from dietary restriction or enzyme replacement therapy would also be helpful. IBS patients who do not improve on a low FODMAP diet may benefit from enzyme replacement therapy, but there are no known distinguishing characteristics between IBS and disaccharidase deficiency. Increased screening and testing along with detailed characterization of the phenotypes will likely help us to learn more about this unique and often misunderstood condition and provide relief for many patients.
